# Toxigenomic Evaluation of Diallyl Disulfide Effects and Its Association with the Chemotherapeutic Agent 5-Fluorouracil in Colorectal Cancer Cell Lines

**DOI:** 10.3390/nu17152412

**Published:** 2025-07-24

**Authors:** Estefani Maria Treviso, Caroline Andolfato Sanchez, Cecília Cristina Souza Rocha, Alexandre Ferro Aissa, Lusânia Maria Greggi Antunes

**Affiliations:** 1 Department of Clinical Analysis, Toxicology Food Science, School of Pharmaceutical Sciences of Ribeirão Preto, University of São Paulo, Ribeirão Preto 14040-903, Brazil; estefanitreviso@usp.br (E.M.T.); carolinesanchez.a@gmail.com (C.A.S.); csrocha@usp.br (C.C.S.R.); 2 Department of Morphology and Genetics, Federal University of São Paulo, São Paulo 04021-001, Brazil; afaissa@gmail.com

**Keywords:** nutraceutical, DNA methylation, chemosensitization, nutrigenomics, comet assay, epigenetics

## Abstract

**Background/Objectives**: Colorectal cancer (CRC) is among the most prevalent malignant neoplasms globally. Chemotherapeutic treatment strategies have demonstrated minimal improvement over the past decade. Combination therapies, including those with nutraceuticals, are currently being investigated as promising alternatives to enhance therapeutic efficacy. The organosulfur garlic extract diallyl disulfide (DADS) has demonstrated anti-tumoral activity in several types of cancer. This study aimed to investigate the effects of DADS and 5-fluorouracil (5-FU), both individually and in combination, on the human CRC cell lines Caco-2 and HT-29. **Methods**: Caco-2, HT-29, and non-tumoral human umbilical vein endothelial cells (HUVEC) were exposed to DADS (25–600 µM) and 5-FU (5–100 µM), either individually or in simultaneous combination (DADS 100 µM + 5-FU 100 µM), for 24 h. Cytotoxicity was evaluated in all three cell lines. In addition, the effects of these treatments on oxidative stress, cell migration, genotoxicity, cell death, global DNA methylation, and gene–nutraceutical interactions were assessed in both tumor cell lines. **Results**: DADS demonstrated cytotoxic effects at high concentrations in Caco-2, HT-29, and HUVECs and induced DNA damage in both colorectal cancer cell lines. The combination of DADS and 5-FU significantly promoted apoptotic cell death, increased genotoxicity, elevated global DNA methylation, and inhibited cell migration, with these effects being particularly pronounced in HT-29 cells. **Conclusions**: We provide evidence that DADS combined with 5-FU is potentially useful in the therapy of CRC. However the combination of nutraceuticals and chemotherapy must consider the distinct molecular and phenotypic characteristics of each tumor cell line.

## 1. Introduction

Colorectal cancer (CRC) is the second most commonly diagnosed cancer and the second leading cause of cancer-related deaths worldwide [1]. It remains largely asymptomatic until it reaches advanced stages. Treatment options include endoscopic and surgical local excision, preoperative downstaging radiotherapy, systemic therapy, extensive surgery for locoregional and metastatic disease, and chemotherapy [2]. Chemotherapeutic intervention, combined with surgery, remains the cornerstone of metastatic CRC treatment and is the only approach that significantly improves survival. For decades after its discovery, the antimetabolite 5-fluorouracil (5-FU) was the only chemotherapeutic agent shown to improve survival in CRC patients [3]. CRC is associated with the overall health of the colon and rectum and is partially influenced by dietary habits [4]. The role of nutrition in colon cancer has been extensively studied, with evidence supporting both causal and protective dietary factors in its development [5]. Potentially protective foods include garlic, magnesium, fish, and vitamin B6 [6].

5-FU is an antimetabolic agent used in the treatment of several types of tumors, including those of the breast, skin, stomach, head, and neck, as well as the colon and rectum [7,8,9,10]. Its antitumor effects are exerted mainly through the inhibition of thymidylate synthase, which disrupts intracellular deoxynucleotide pools necessary for DNA replication. Additional mechanisms include incorporation into RNA, resulting in disrupted RNA synthesis, and incorporation into DNA, leading to DNA fragmentation. It has been demonstrated that only up to 3% of the original 5-FU dose exerts cytotoxic effects on tumor and non-malignant cells through these anabolic actions. The vast majority of 5-FU is catabolized into inactive metabolites by dihydropyrimidine dehydrogenase, an enzyme abundantly expressed in the liver [11].

The resistance of tumor cells to chemotherapeutic drugs is one of the leading causes of chemotherapy failure. Resistance to 5-FU, in particular, is a major factor contributing to the unsuccessful treatment of CRC. Nonetheless, the combination of chemotherapy with chemosensitizing agents may provide a synergistic therapeutic effect, reduce toxicity, and delay the development of drug resistance [12]. 5-FU is widely used in combination with other agents, such as oxaliplatin, to treat cancer progression and metastasis [13].

The nutraceutical market is currently a high-impact, multi-billion-dollar industry, with projections indicating rapid growth over the next decade. Nutraceuticals comprise a diverse array of food-derived products that have gained popularity owing to increased consumer awareness of their potential health benefits and the rising demand for enhanced wellness [14]. Compounds present in garlic extracts—especially those exhibiting anticancer properties—are supported by both clinical and laboratory data, suggesting their capacity to potentiate the efficacy of 5-FU in CRC treatment [15]. The organosulfur compound derived from garlic, diallyl disulfide (DADS), has been demonstrated to induce cell cycle arrest and apoptosis and to exert anti-proliferative effects across various CRC cell lines [16]. A previous study demonstrated that the combination of DADS and Sorafenib inhibited cell proliferation, migration, and invasion in hepatocellular carcinoma cells [17]. Given the promising results reported for DADS, this study aimed to evaluate its effects, both alone and in combination with 5-FU, on the colorectal adenocarcinoma cell lines Caco-2 and HT-29.

## 2. Materials and Methods

### 2.1. Cell Culture

The human CRC cell lines Caco-2 (BCRJ code: 0059) and HT-29 (BCRJ code: 0111) were obtained from the Rio de Janeiro Cell Bank (Brazil), while human umbilical vein endothelial cells (HUVEC; CRL-1730) were obtained from the American Type Culture Collection. All cell lines were cultured in low-glucose Dulbecco’s modified eagle medium, pH 7.4, supplemented with 100× penicillin-streptomycin solution antibiotic solution, 3.7 g/L sodium bicarbonate, and fetal bovine serum at a concentration of 10% for HT-29 and HUVEC and 20% for Caco-2. Cells were maintained in an incubator at 37 °C with 5% CO_2_ and 95% humidity. Subculturing and maintenance protocols adhered to the guidelines outlined in the Guidance on Good Cell Culture Practice [18]. All experiments were conducted using cells between passages 3 and 8 post-thaw.

### 2.2. Reagents

Diallyl disulfide (CAS 2179-57-9), 5-FU (CAS 51-21-8), resazurin (CAS 62758-13-8), trypan blue (CAS 72-57-1), dimethyl sulfoxide (DMSO; CAS D5879), methyl methanesulfonate (CAS 66-27-3), and doxorubicin (CAS 25316-40-9) were obtained from Sigma-Aldrich (Saint Louis, MO, USA). Dulbecco’s modified eagle medium (Gibco ref. 31600-034), fetal bovine serum (Gibco ref. 12657029), 100× penicillin-streptomycin solution (Gibco ref. 15640-055), and TrypLE Express (Gibco ref. 12604-021) were purchased from Thermo Fisher Scientific (Waltham, MA, USA).

### 2.3. Treatment Protocol

Stock solutions of DADS and 5-FU were prepared by dissolving each compound separately in DMSO. Immediately before treatment, DADS and 5-FU were further diluted in a complete culture medium to their final concentrations. After the seeding incubation period, cells were treated for 24 h with DADS, 5-FU, or their simultaneous combination. The culture medium served as the negative control, while 0.25% DMSO was used as the solvent control. Positive controls were included according to the requirements of each assay: 300 µM MMS for the cell viability and migration assays, 400 µM MMS for the comet assay, 1 mM H_2_O_2_ for reactive oxygen species detection, and 2 µM doxorubicin for apoptosis analysis. All assays were performed in biological triplicate.

### 2.4. Cell Viability

Cell viability was assessed following a protocol adapted from Page and collaborators [19]. Cells were seeded in 96-well plates at a density of 1 × 10^4^ cells per well and incubated for 24 h prior to the treatments. After the treatment period, the medium was removed, and cells were washed with phosphate-buffered saline (PBS). Subsequently, 15 µL of resazurin (398 µM) diluted in 85 µL of culture medium was added to each well. Plates were incubated for 4 h at 37 °C. Fluorescence was then measured using a Synergy 2 microplate reader (BioTek, Charlotte, VT, USA) at excitation and emission wavelengths of 530 and 590 nm, respectively. Cell viability was expressed as a percentage relative to the negative control, which was defined as 100% viable cells.

### 2.5. Reactive Species

Reactive species (RS) levels were determined using the CM-H_2_DCFDA probe (Life Technologies, Carlsbad, CA, USA), following the manufacturer’s instructions. In summary, the probe was dissolved in 34.6 µL of 99% ethanol and diluted in 17 mL of PBS to a final concentration of 5 µM. Cells were seeded at 1 × 10^4^ cells per well in black-walled, clear-bottom 96-well plates and incubated for 24 h, followed by treatment as described above. Following treatment, the culture medium was removed and cells were washed with PBS. Subsequently, 100 µL of CM-H_2_DCFDA (5 µM) was added to each well. Plates were incubated for 50 min at 37 °C in the dark. For the positive controls, 20 µL of 1 mM H_2_O_2_ was added to the designated wells 20 min before the end of the incubation period. After incubation, the probe was removed and 100 µL of PBS was added to each well. Fluorescence was measured using a Synergy 2 microplate reader (BioTek, Charlotte, VT, USA) with excitation and emission wavelengths set at 450 and 520 nm, respectively. Reactive species levels were expressed as a percentage relative to the negative control.

### 2.6. Cell Migration

Cell migration was evaluated using the wound healing assay. A total of 2.5 × 10^4^ cells were seeded in 24-well plates and incubated for 24 h. Following incubation, the culture medium was removed, and a scratch was made in the cell monolayer using a 100 µL pipette tip. Wells were washed with PBS to remove detached cells from the scratched area. Treatments were prepared in a culture medium supplemented with 2% fetal bovine serum. Images were acquired at 0 and 24 h of incubation using a camera mounted on an inverted microscope (Zeiss Primovert, Jena, Germany). The scratch area was quantified using Fiji (ImageJ) x86-64 software. The wound closure (%) was calculated based on the reduction in wound area relative to the negative control.

### 2.7. Genotoxicity

Cells were seeded at a density of 2.5 × 10^4^ cells per well in 24-well plates and incubated for 24 h, followed by treatment as previously described. After treatment, cell viability was assessed using the trypan blue exclusion assay. Only samples demonstrating at least 70% viability were selected for the comet assay, which was conducted under alkaline conditions. Microscope slides were pre-coated with 1.5% normal melting point agarose and allowed to solidify. Subsequently, 60 µL of a homogenate containing cells and 0.5% low melting point agarose was layered onto the pre-coated slides, covered with a coverslip, and incubated at 4 °C for 20 min. Coverslips were then removed, and the slides immersed in a lysis solution (2.5 M NaCl, 100 mM ethylenediamine tetraacetic acid, 10 mM Tris, 1% Triton X-100, and 10% DMSO; pH 10) overnight at 4 °C in the dark.

Following lysis, the slides were gently washed with distilled water and subjected to electrophoresis in an ice-cold alkaline buffer (300 mM NaOH, 1 mM ethylenediamine tetraacetic acid; pH > 13) at a constant voltage of 25 V and a current of 300 mA for 20 min. Subsequently, the slides were neutralized in 0.4 M Tris buffer (pH 7.5) for 5 min, air-dried at room temperature, and fixed in absolute ethanol for 5 min. Immediately before analysis, slides were stained with GelRed (1:10,000 in PBS) for 3 min. Nucleoids were visualized using a fluorescence microscope (Zeiss AxioStar Plus, Jena, Germany) equipped with a 20× objective, an excitation filter of 515–560 nm, and an emission barrier filter of 590 nm. For each treatment, 100 nucleoids were analyzed using CometAssay IV 4.3 software (Perceptive Instruments, Haverhill Suffol, UK). The percentage of DNA in the comet tail (tail intensity) was used as the primary parameter for DNA damage quantification.

### 2.8. Apoptosis

Apoptotic cells were identified using the Alexa Fluor 488 Annexin V apoptosis detection kit (Annexin V-FITC, Thermo Fisher Scientific, Waltham, MA, USA), following the manufacturer’s instructions. In short, 2.5 × 10^4^ cells were seeded in 24-well plates and incubated for 24 h to ensure adherence. After incubation, cells were treated as previously described, with 2 µM doxorubicin included as positive control. Following treatment, cells were detached using Accutase, centrifuged, and gently resuspended in 1× Annexin binding buffer. Cells were then stained with Annexin V-FITC and incubated on ice for 15 min. Propidium iodide (PI) was added immediately before flow cytometric analysis.

Samples were analyzed on a BD LSRFortessa flow cytometer (BD Bioscience, Franklin Lakes, NJ, USA) at excitation/emission wavelengths of 488/520 nm. A total of 20,000 events were recorded per sample. The percentages of apoptotic and necrotic cells were determined based on Annexin V-FITC and PI staining. Quadrant Q1 represented necrotic cells (Annexin V^−^/PI^+^), Q2 and Q4 corresponded to early and late apoptotic cells (Annexin V^+^/PI^−^ and Annexin V^+^/PI^+^, respectively), and Q3 represented viable cells (Annexin V^−^/PI^−^). Total apoptosis was calculated as the sum of the percentages in Q2 and Q4.

### 2.9. Global DNA Methylation

Genomic DNA was isolated using the Wizard Genomic DNA Purification Kit (Promega, Madison, WI, USA) according to the manufacturer’s instructions. Global DNA methylation was quantified with the MethylFlash Methylated 5mC DNA Quantification Kit (P-1034, Epigentek, Farmingdale, NY, USA) following the manufacturer’s protocol. Absorbance was measured at 450 nm using a microplate reader. The percentage of methylated DNA was calculated relative to the control using the formula provided with the kit.

### 2.10. Protein Expression

Protein expression was assessed by Western blot analysis. Cells were seeded in 100 mm culture dishes at a density of 2 × 10^6^ cells and incubated for 24 h; after incubation, treatments were performed as previously described. Cells were washed with ice-cold PBS (pH 7.4), and total proteins were extracted using 1× RIPA buffer (Abcam, ab156034, Cambridge, UK) supplemented with protease (P8340, Sigma-Aldrich, St. Louis, MO, USA) and phosphatase (78420, Thermo Fisher Scientific, Waltham, MA, USA) inhibitors. Cell lysates were centrifuged at 10,000× *g* for 15 min at 4 °C, and the supernatants were collected. Protein concentration was determined using the Pierce BCA Protein Assay Kit (23227, Thermo Fisher Scientific, Waltham, MA, USA). All steps were carried out on ice under cold conditions. Samples were stored at −80 °C until further analysis. For electrophoresis, 30 µg of total protein was mixed with 4× SDS loading buffer (containing glycerol, 0.5 M Tris-HCl, sodium dodecyl sulfate, and bromophenol blue) and β-mercaptoethanol, then heat-denatured at 90 °C for 5 min. Samples were separated on a homemade bisacrylamide gel by SDS-PAGE and transferred onto nitrocellulose membranes. Membranes were blocked in 5% bovine serum albumin for 20 min at room temperature and incubated overnight at 4 °C with the following primary antibodies: anti-DNMT1 [EPR18453] (Abcam, ab188453; 1:1000, Cambridge, UK), anti-VEGFA [EPR20705] (Abcam, ab214424; 1:1000, Cambridge, UK), and glyceraldehyde-3-phosphate dehydrogenase (GAPDH; D16H11, Cell Signaling, Danvers, MA, USA, 5174S; 1:1000) as a loading control. After primary antibody incubation, membranes were washed with Tris-buffered saline with Tween 20 and then incubated with horseradish peroxidase-conjugated secondary antibody (goat anti-rabbit IgG [H + L], Abclonal, Woburn, MA, USA, AS014; 1:10,000). Following additional washes, the detection was performed using the ChemiDoc MP Imaging System (Bio-Rad, Hercules, CA, USA), and bands were analyzed with Image Lab 6.1 software (Bio-Rad, Hercules, CA, USA). Protein expression was reported as a relative expression normalized to GAPDH. The experiments were performed in biological triplicates.

### 2.11. Statistical Analysis

Statistical analysis was conducted using GraphPad Prism version 8.0 (GraphPad Software, Boston, MA, USA), including calculations of half-maximal inhibitory concentration (IC_50_) by nonlinear regression. The Shapiro–Wilk test was used to evaluate data normality. For comparisons among multiple groups, a one-way analysis of variance (ANOVA) followed by Tukey’s post hoc test was applied.

## 3. Results

### 3.1. Cell Viability

#### 3.1.1. Isolate Treatments

**Figure 1:**
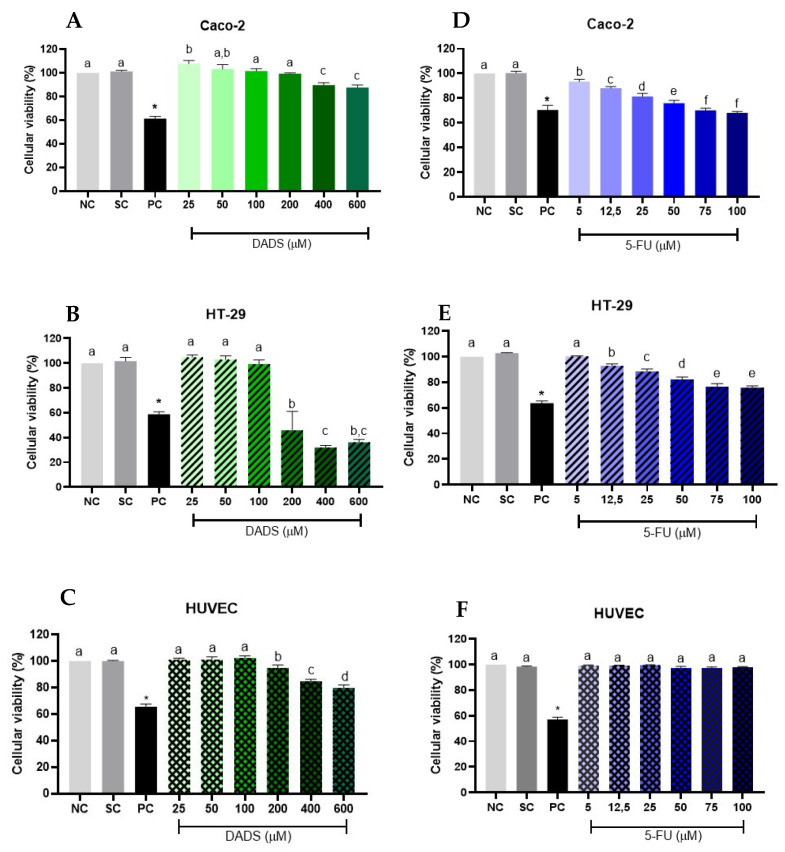
Cell viability was evaluated using the resazurin assay following 24 h treatments with diallyl disulfide (DADS; 25–600 µM) in Caco-2 (**A**), HT-29 (**B**), and HUVECs (**C**), and with 5-fluorouracil (5–100 µM) in Caco-2 (**D**), HT-29 (**E**), and HUVECs (**F**). Negative control (NC): culture medium only; solvent control (SC): 0.25% dimethyl sulfoxide; positive control (PC): 300 µM methyl methanesulfonate. Data are presented as mean ± standard deviation (*n* = 3). Statistical analysis was conducted using one-way ANOVA followed by Tukey’s post hoc test. Columns with the same letter are not significantly different (*p* > 0.05). * Significant difference compared to the negative control (Student’s *t*-test, *p* < 0.05).

Caco-2 cells exposed to DADS (25–600 µM) for 24 h only exhibited decreased cell viability at 400 and 600 µM, with reductions of 11 and 13%, respectively, compared to the negative control (Figure 1A). In contrast, HT-29 cells showed lower viability, with 46% viability at 200 µM, 31% at 400 µM, and 36% at 600 µM DADS after 24 h of exposure (Figure 1B). For 5-FU treatment (5–100 µM), Caco-2 cells exhibited progressively lower viability across all tested concentrations after 24 h (Figure 1D). HT-29 cells also demonstrated reduced viability, with approximately 76% viability at 100 µM (Figure 1E).

The non-tumoral HUVEC cell line displayed a marked decrease in viability in response to DADS, with reductions of 6, 15, and 21% at 200, 400, and 600 µM, respectively, after 24 h of treatment (Figure 1C). Conversely, HUVECs exposed to 5-FU (5–100 µM) for 24 h did not exhibit significant changes in viability at any tested concentration compared to the negative control (Figure 1F).

#### 3.1.2. Associated Treatments

**Figure 2:**
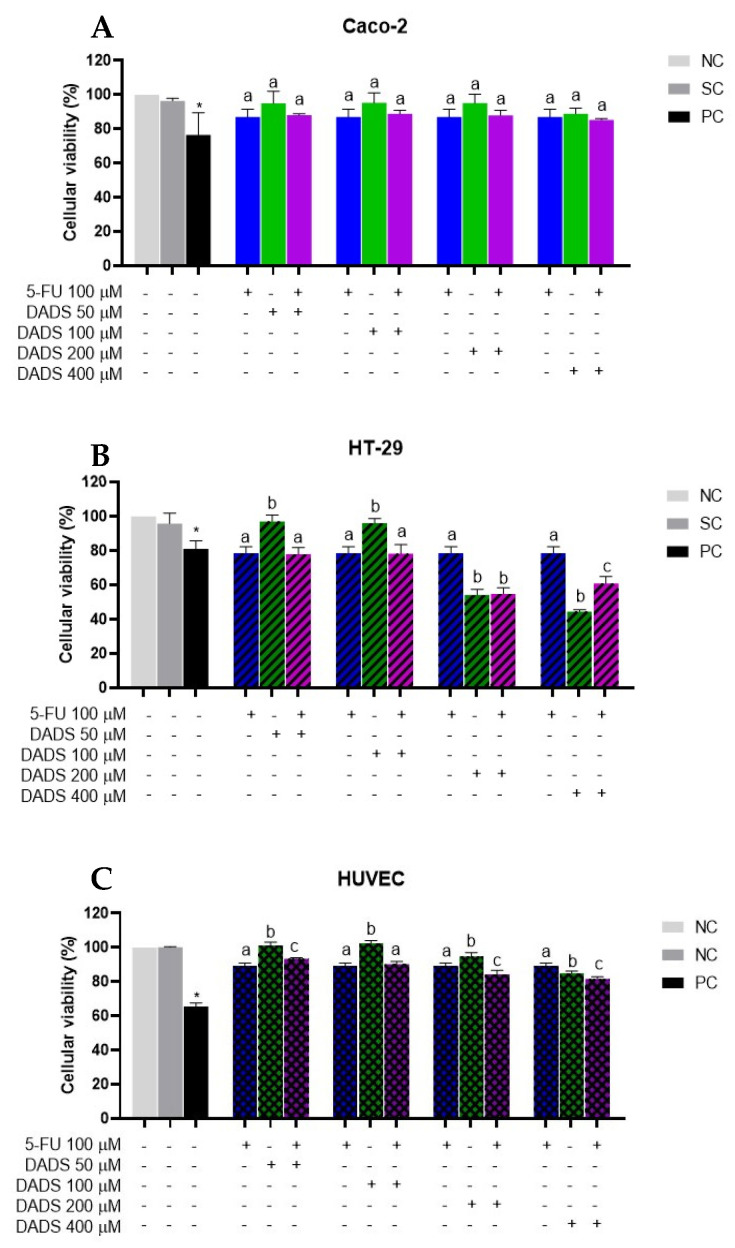
Cell viability was assessed using the resazurin assay after a 24 h simultaneous treatment with diallyl disulfide (DADS; 50–400 µM) and 5-fluorouracil (5-FU; 100 µM) in Caco-2 (**A**), HT-29 (**B**), and HUVECs (**C**). Negative control (NC): culture medium only; solvent control (SC): 0.25% dimethyl sulfoxide; positive control (PC): 300 µM methyl methanesulfonate. Results are presented as mean ± standard deviation (*n* = 3). Statistical significance was determined using one-way ANOVA followed by Tukey’s post hoc test. Columns with the same letter are not significantly different (*p* > 0.05). * Significant difference compared to the negative control (Student’s *t*-test, *p* < 0.05).

No significant changes in cell viability were observed in Caco-2 cells following combined treatment with DADS (50–400 µM) and 5-FU (100 µM) (Figure 2A). In contrast, HT-29 cells exhibited a reduction in viability of approximately 35% at the two highest concentrations of DADS alone. The co-administration of DADS (200 µM) and 5-FU (100 µM) resulted in approximately 60% viability. Similarly, HT-29 cells exposed to DADS (400 µM) in combination with 5-FU (100 µM) demonstrated a viability of approximately 70% (Figure 2B). In HUVECs, co-treatment with 100 µM 5-FU in combination with DADS at 200 and 400 µM decreased cell viability (Figure 2C).

### 3.2. Reactive Species

**Figure 3:**
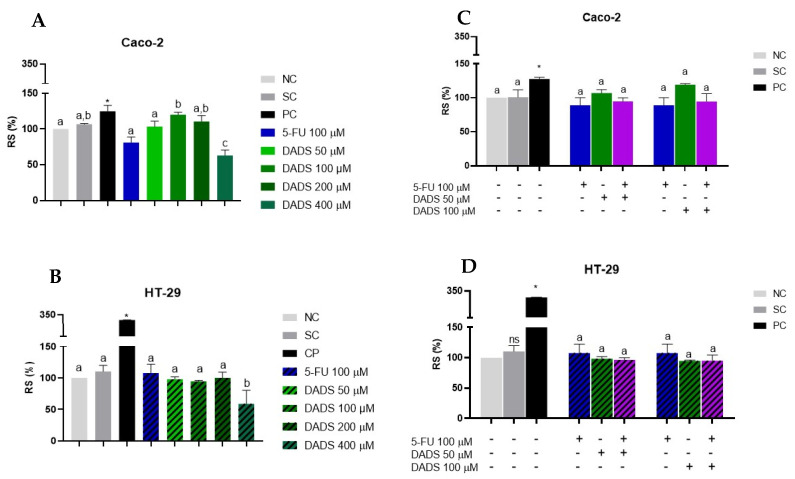
Analysis of reactive species (RS) generation after treatment with diallyl disulfide (DADS) and 5-fluorouracil (5-FU) in colorectal cancer cells. Isolated treatments with DADS (50–400 µM) and 5-FU (100 µM) in Caco-2 (**A**) and HT-29 (**B**) cells, as well as combined treatments with DADS (50 and 100 µM) and 5-FU (100 µM) in Caco-2 (**C**) and HT-29 (**D**) cells, were assessed after 24 h of exposure. Negative control (NC): culture medium; solvent control (SC): 0.25% dimethyl sulfoxide; positive control (PC): 1 mM hydrogen peroxide (H_2_O_2_). Data are presented as mean ± standard deviation (*n* = 3). Statistical significance was assessed using one-way ANOVA followed by Tukey’s post hoc test. Bars sharing the same letter do not differ significantly (*p* > 0.05). * Significant difference compared to the negative control (Student’s *t*-test, *p* < 0.05).

Caco-2 cells exhibited a significant reduction in RS generation following treatment with DADS at 400 µM (Figure 3A). However, treatment with 5-FU (100 µM) alone did not alter RS levels in either cell line (Figure 3A,B). In HT-29 cells, a significant decrease in RS production was observed only with DADS at 400 µM. Combined treatment with 5-FU (100 µM) and DADS (50 or 100 µM) did not result in significant differences in RS levels compared to the respective individual treatments in either Caco-2 or HT-29 (Figure 3C,D).

### 3.3. Cell Migration

**Figure 4:**
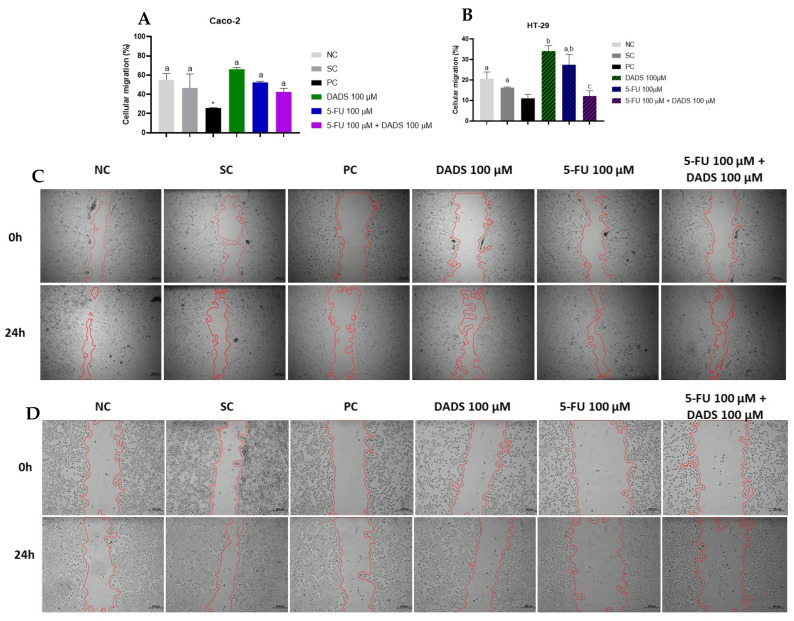
Evaluation of relative cell migration by wound healing assay after treatment with diallyl disulfide (DADS, 100 µM), 5-fluorouracil (5-FU, 100 µM), and their combination over 24 h. Wound closure (%) was calculated by comparing the reduction in the wound area to that of the negative control. (**A**) Quantification of wound closure in Caco-2 and (**B**) HT-29 cells. (**C**,**D**) Representative photomicrographs of wound healing at 0 and 24 h in Caco-2 and HT-29 cells, respectively. Negative control (NC): culture medium; solvent control (SC): 0.25% dimethyl sulfoxide; positive control (PC): 300 µM methyl methanesulfonate. Data are presented as mean ± standard deviation (*n* = 3). Statistical analysis was conducted using one-way ANOVA followed by Tukey’s post hoc test. Bars with identical letters are not significantly different (*p* > 0.05). * Statistically significant difference compared to the negative control (Student’s *t*-test, *p* < 0.05).

Caco-2 cells did not exhibit significant changes in cell migration following any treatment after 24 h (Figure 4A). Conversely, in HT-29 cells, the combined treatment significantly inhibited migration, reducing wound closure to approximately 12%. Isolated treatment with DADS enhanced migration, resulting in about 34% wound closure. HT-29 cells treated with 5-FU alone showed no significant differences compared to the negative control (Figure 4B).

Representative images from the wound healing assay for Caco-2 (Figure 4C) and HT-29 (Figure 4D) cells at 0 and 24 h demonstrate the effects of the various treatments on cell migration.

### 3.4. Cell Death and Apoptosis

**Figure 5:**
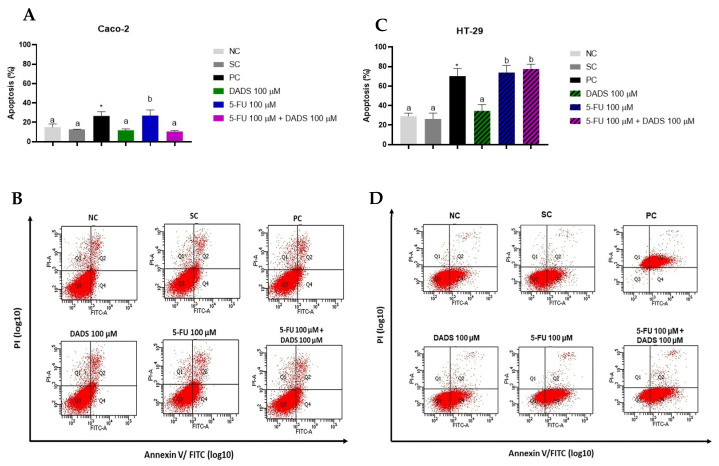
Characterization of the cell death profile in Caco-2 and HT-29 cells after 24 h of exposure to diallyl disulfide (DADS, 100 µM) and 5-fluorouracil (5-FU, 100 µM), administered both individually and in combination. (**A**,**C**) Quantification of apoptotic and necrotic cells in Caco-2 and HT-29 cells, respectively. (**B**,**D**) Representative dot plots showing Annexin V-FITC/PI staining distribution: Q3—viable cells (double-negative); Q4—early apoptotic (Annexin V-FITC positive only); Q1—necrotic (PI positive only); Q2—late apoptotic or secondary necrotic (double-positive). The negative control (NC) consisted of the culture medium; the solvent control (SC) was 0.25% dimethyl sulfoxide; and the positive control (PC) was 2 µM doxorubicin. Data are presented as mean ± standard deviation (*n* = 3). Statistical analysis was carried out using one-way ANOVA followed by Tukey’s post hoc test. Columns with the same letter are not significantly different (*p* > 0.05). * Statistical significance compared to the negative control (Student’s *t*-test, *p* < 0.05).

In Caco-2 cells, treatment with 5-FU alone effectively induced apoptosis. However, DADS alone or in combination with 5-FU did not result in significant differences in apoptosis compared to the negative control (Figure 5A). In HT-29 cells, exposure to 100 µM of 5-FU, both alone and in combination with 100 µM of DADS, significantly induced apoptosis. The levels of apoptosis observed in the group treated with both 5-FU and DADS were not significantly different from those induced by 5-FU alone. DADS at a concentration of 100 µM did not significantly affect apoptosis in HT-29 cells (Figure 5C).

### 3.5. Genotoxicity

**Figure 6:**
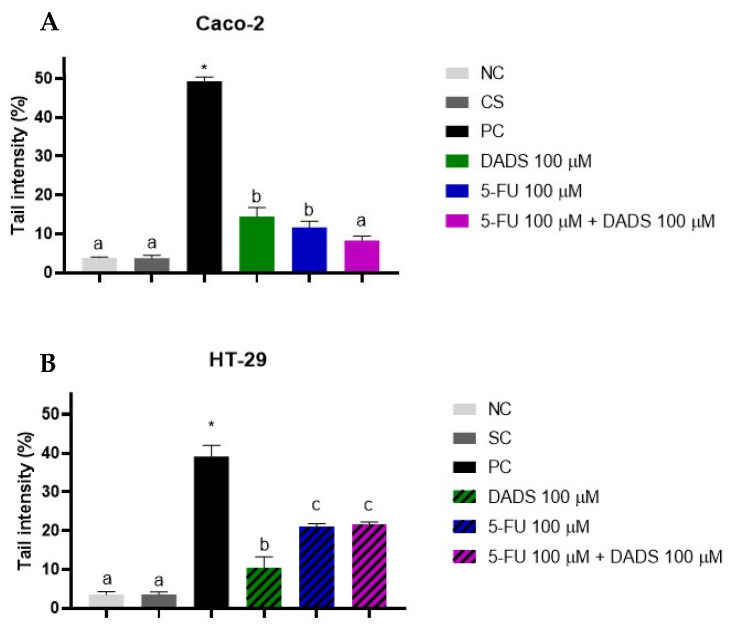
Quantification of DNA damage using the comet assay after a 24 h treatment with diallyl disulfide (DADS, 100 μM), 5-fluorouracil (5-FU, 100 μM), and their combination in Caco-2 (**A**) and HT-29 (**B**) cells. DNA damage is expressed as the percentage of DNA in the comet tail. Negative control (NC): culture medium; solvent control (SC): 0.25% dimethyl sulfoxide; positive control (PC): 400 µM methyl methanesulfonate. Data are presented as the mean ± standard deviation (*n* = 3). Statistical analyses were performed using one-way ANOVA followed by Tukey’s post hoc test. Columns with the same letter are not significantly different (*p* > 0.05). * Statistical significance compared to the negative control (Student’s *t*-test, *p* < 0.05).

Genotoxicity assessment revealed that both DADS (100 µM) and 5-FU (100 µM), when used as individual treatments, induced DNA damage in the Caco-2 cell line relative to the negative control. Nonetheless, combined treatment with DADS and 5-FU (100 µM each) did not increase DNA damage beyond levels observed with the negative control in this cell line (Figure 6A). In the HT-29 cell line, all treatments resulted in DNA damage. Hence, the combination of DADS (100 µM) and 5-FU (100 µM) resulted in greater DNA damage compared to the effect of DADS alone, although this increase was not statistically significant when compared to the 5-FU treatment alone (Figure 6B).

### 3.6. Global DNA Methylation

**Figure 7:**
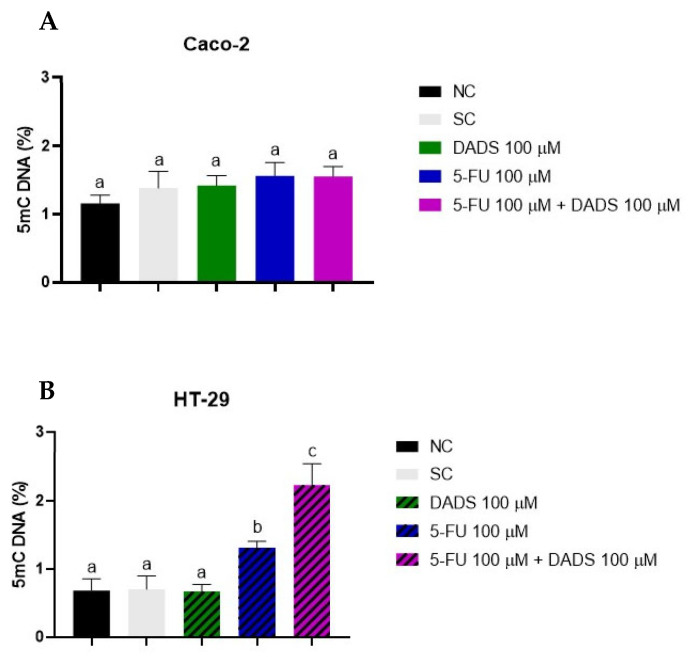
Methylated DNA content after treatment with diallyl disulfide (DADS, 100 µM), 5-fluorouracil (5-FU, 100 μM), and their combination in Caco-2 (**A**) and HT-29 (**B**) cells. Negative control (NC): culture medium; solvent control (SC): 0.25% dimethyl sulfoxide. The data are presented as the mean ± standard deviation (*n* = 3). Statistical analysis was performed using one-way ANOVA followed by Tukey’s post hoc test. Columns with the same letter are not significantly different (*p* > 0.05).

Caco-2 cells did not exhibit significant changes in global DNA methylation levels following any of the treatments tested (Figure 7A). In HT-29 cells, treatment with DADS alone did not significantly alter DNA methylation compared to the control. Nevertheless, treatment with 5-FU alone significantly increased global DNA methylation. The combined treatment with DADS and 5-FU resulted in a markedly higher level of global DNA methylation than either treatment alone (Figure 7B).

### 3.7. Protein Expression

**Figure 8:**
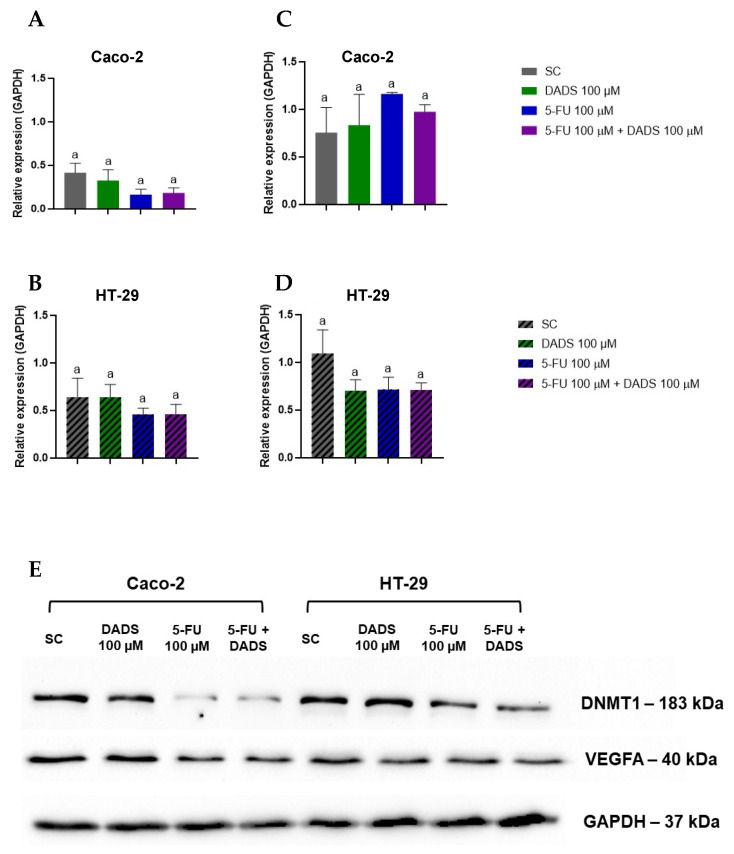
Relative protein expression of DNMT1 in Caco-2 (**A**) and HT-29 (**B**) and VEGFA in Caco-2 (**C**) and HT-29 (**D**) following treatment with diallyl disulfide (DADS, 100 µM), 5-fluorouracil (5-FU, 100 µM), and their simultaneous exposure for 24 h. (**E**) Representative image of the lanes. Data are presented as mean ± standard deviation (*n* = 3). Statistical analysis was performed using one-way ANOVA followed by Tukey’s post hoc test. Columns with the same letter are not significantly different (*p* < 0.05).

We assessed the relative expression levels of DNA methyltransferase 1 (DNMT1) and vascular endothelial growth factor A (VEGFA) in Caco-2 and HT-29 cells. In Caco-2 cells, DNMT1 and VEGFA expression remained unchanged under all treatment conditions (Figure 8A,C), and this was also the case in HT-29 cells (Figure 8B,D). Western blot images demonstrating the protein levels of DNMT1 (183 kDa), VEGFA (40 kDa), and GAPDH (37 kDa; serving as a loading control) in Caco-2 and HT-29 cells are shown in Figure 8E.

## 4. Discussion

Drug resistance and adverse effects represent significant challenges in the treatment of CRC. One strategy to address these issues is the use of natural products in combination with conventional chemotherapeutic agents, such as 5-FU, to sensitize tumor cells and enhance the efficacy of chemotherapy in CRC [13]. In this study, we investigated the effects of DADS, both alone and in combination with 5-FU, on the human CRC cell lines Caco-2 and HT-29.

The Caco-2 and HT-29 cells are widely recognized as suitable models for studying intestinal absorption and xenobiotic toxicity. Nevertheless, research has indicated that the expression of various proteins, including transporters, differs in these cell lines compared to human intestinal tissues [20]. Despite these differences, Mouradov et al. [21] analyzed mutation profiles, microarray data, and exome sequences from 70 CRC cell lines, concluding that these models represent the major molecular subtypes of primary colorectal tumors. Therefore, Caco-2 and HT-29 remain valuable models for investigating CRC biology and responses to therapeutic agents [21].

Berg and collaborators [22] analyzed integrated DNA, RNA, and protein data from 34 human CRC cell lines. In their study, 612 genes were sequenced, and the expression of 297 proteins was assessed. The authors identified consistent molecular subgroups based on genomic instability, sequencing data, and the differential expression of mRNA, miRNA, and protein levels. Two distinct clusters emerged: cell lines with a colon-like phenotype, typified by the expression of gastrointestinal differentiation markers, and undifferentiated cell lines, characterized by upregulation of epithelial–mesenchymal transition factors. HT-29 cells were classified as colon-like, while Caco-2 cells were categorized as undifferentiated [22]. According to the same authors, undifferentiated cell lines such as Caco-2 exhibit higher expression of epithelial–mesenchymal transition-related genes and are associated with increased metastatic potential. Thus, Caco-2 and HT-29 cells provide valuable models for evaluating the effects of compounds, including DADS and 5-FU, given their distinct molecular profiles and differential responses to treatment.

The concentrations of DADS (25–600 µM) and 5-FU (5–100 µM) were selected based on prior literature and results from our group [17,23,24,25]. Vodenkova and coauthors [11] showed that most 5-FU is metabolized within the first 24 h in the human body. Although pharmacokinetic data for DADS in humans are lacking, rodent studies have demonstrated that maximal plasma concentrations are achieved within one day of oral administration [26]. Therefore, we defined 24 h as the optimal treatment duration.

Our results indicated that DADS, when administered alone, was cytotoxic at the highest concentrations tested (400–600 µM) in the Caco-2, HT-29, and non-tumorigenic HUVEC cell lines. Altonsy and Andrews [27] found that DADS could induce cell cycle arrest in the G2/M phase in HT-29 cells, independent of concentration. Similarly, they observed a 10% reduction in cell number following exposure to 100 µM DADS for 24 h. Huang et al. [28] reported that DADS inhibited cell proliferation in a time- and concentration-dependent manner in HT-29 cells, whereas Machado and collaborators [17] noted that DADS reduced cell viability by 20–30% at 100 and 400 µM in HepG2 cells. These findings suggest that the effects of DADS may vary depending on the cell type examined.

Exposure to 5-FU (5–100 µM) significantly decreased cell viability at nearly all tested concentrations in Caco-2 and HT-29 cells, which corroborates the findings of Onar et al. and Yildiz et al. [29,30], who found that 5-FU reduced cell viability in Caco-2 and HT-29 cells after 24 h of exposure. In theHT-29 cell line, cytotoxicity was observed at DADS concentrations of 200 and 400 µM combined with 5-FU, although no synergistic effects were noted. Machado et al. [17] reported cytotoxicity to HepG2 cells at DADS at 50, 100, and 200 µM, and noted increased cytotoxicity when DADS was combined with 8 µM sorafenib.

Thejass and Kuttan [31] observed a decrease in HUVEC cell viability after exposure to DADS at concentrations ranging from 6.9 to 347.8 µM for 48 h. Rashidi et al. [32] reported that HUVECs showed approximately a 10% reduction in cell viability at concentrations above 20 µM when treated with 5-FU for 24 h, albeit without a clear dose–response relationship. In our study, 5-FU did not significantly affect HUVEC viability at the tested concentrations.

Reactive species (RS) are generated as byproducts of various intracellular processes, including cellular respiration and exposure to xenobiotics. In Caco-2 and HT-29 cells treated with a combination of DADS and 5-FU for 24 h, no significant increase in RS production was observed. The reduction observed in RS production at higher concentrations of DADS alone in both cell lines ispossibly due to decreased cell viability resulting in less RS generation. 

Wu et al. [33] observed an increase in RS production induced by DADS in A549 lung cells, while Song et al. [34] demonstrated that RS production increased during the first 30 min of treatment, peaking at 1.5 h with 200 µM DADS, then rapidly declining after 3 h in HCT116 CRC cells. After 24 h, the cells could have restored their intracellular redox balance, potentially explaining the absence of RS detection.

Anticancer agents that inhibit migration may enhance therapeutic effectiveness [35]. In our study, treatments with isolated DADS, 5-FU, and their combination did not alter cell migration in Caco-2 cells. However, the combination of DADS and 5-FU inhibited cell migration in HT-29 cells. 

The relationship between VEGFA expression and cell migration was investigated through Western blot analysis. In Caco-2 cells, no significant changes in cell migration or VEGFA expression were observed across different treatment groups. In HT-29 cells, co-treated with DADS and 5-FU, significant migration inhibition was observed without altering VEGFA expression. 

Genotoxicity was assessed using the comet assay to observe DNA fragmentation. In the Caco-2 cells, the inductions o DNA damage was observed in DADS e 5-FU alone, but not in the combination. In contrast, HT-29 cell line presented DNA damage in all treatments. Despite this, the accumulation of DNA damage with the combined treatment did not differ from that caused by 5-FU alone, suggesting that, in HT-29 cells, the genotoxic effects are primarily attributed to 5-FU’s mechanism. The results in Caco-2 cells suggest that DADS may exert a protective action against 5-FU-induced DNA damage, although the underlying mechanism remains unclear. Matuo et al. [36] identified DNA damage in SW620 CRC cells treated with various concentrations of 5-FU. These authors found that 5-FU interferes with DNA by incorporating nucleotide analogs, inhibiting thymidylate synthase and impairing DNA polymerase’s fidelity. These mechanisms lead to errors during DNA synthesis, nucleotide pool imbalances, blockages of DNA polymerase, and interference with repair mechanisms, contributing to its cytotoxicity.

Apoptosis, a normal physiological response to stimuli such as infection or cellular damage, including that induced by cytotoxic drugs or radiation, which create irreparable DNA lesions [37], was also studied. Isolated 5-FU treatment induced apoptosis, while DADS alone and the combination of DADS with 5-FU did not show significant changes in apoptosis in Caco-2 cells. Conversely, HT-29 cells saw a significant increase in apoptosis with both isolated 5-FU and the combination treatment with DADS. Jakubíková and Sedlák [38] demonstrated increased apoptosis in Caco-2 cells after 48 h of exposure to DADS (50–2000 µM). Altonsy and Andrews [27] reported a 39% increase in apoptosis in HT-29 cells exposed to 100 µM DADS for 24 h. Our results for HT-29 cells differ from those previously reported in the literature regarding the apoptotic response to DADS.

Epigenetic alterations, particularly DNA methylation, contribute to the pathogenesis, molecular heterogeneity, and progression of CRC. Among these, DNA methylation is the most well-characterized epigenetic modification. It involves the covalent addition of a methyl group (CH_3_–) from S-adenosyl methionine to the 5-position of a cytosine base within CpG dinucleotides by DNA methyltransferases (DNMTs), resulting in the formation of 5-methylcytosine (5mC) [39]. Akone et al. [40] identified DADS as a member of the histone deacetylase inhibitor group. Druesne et al. [41] found that treatment with DADS reduces cell proliferation by suppressing histone deacetylase activity, promoting histone hyperacetylation, and increasing p21^Waf1/Cip1^ expression. These findings suggest that the absence of changes in global DNA methylation in Caco-2 cells may be due to DADS exerting its epigenetic effects predominantly through histone acetylation. The HT-29 cell line, however, showed a significant increase in global DNA methylation in response to both individual and combined treatments with DADS and 5-FU, suggesting a cell line-specific epigenetic response. 

The relationship between DNMT1 expression and global DNA methylation levels underscores the complexity of epigenetic regulation in CRC cell lines. In Caco-2 cells, DNMT1 expression was not altered, consistent with the unchanged global DNA methylation levels. Sarabi and Naghibalhossaini [42] stated that global DNA methylation was directly proportional to the expression of DNA methyltransferases, including DNMT1 in Caco-2. This finding suggests that DNMT1 expression alone may not be sufficient to drive widespread methylation changes, possibly due to post-translational modifications or regulatory interactions with other epigenetic factors [43,44]. Additionally, it is plausible that DNMT1 activity, rather than its expression level, is the critical determinant of methylation outcomes [45]. In contrast, HT-29 cells exhibited an increase in global DNA methylation without a change in DNMT1 expression, which may reflect compensatory upregulation of de novo DNA methyltransferases or a shift in the balance between DNA demethylation and remethylation dynamics [46].

The CpG island methylator phenotype (CIMP) defines approximately 30% of CRC cases by widespread hypermethylation of multiple CpG islands in tumor DNA. CIMP-positive (CIMP+) tumors are more frequently associated with a proximal colon location, microsatellite instability, and wild-type TP53 status [47]. Geibler and collaborators [48] classified HT-29 as CIMP+, while Caco-2 was classified as CIMP-. Notably, CIMP+ tumors are generally linked to poorer prognoses, although they may respond better to adjuvant therapies [49]. The molecular heterogeneity observed between CRC cell lines (e.g., Caco-2 and HT-29) may explain the differing outcomes seen in response to treatment.

## 5. Conclusions

This study investigated the toxicogenomic effects of combining DADS with 5-FU on human colorectal adenocarcinoma cell lines. Our findings indicated that DADS alone exerted cytotoxic effects on Caco-2, HT-29, and HUVECs only at higher concentrations. Additionally, DADS induced DNA damage in both tumor cell lines and promoted cell migration in HT-29 cells. The combined use of the nutraceutical DADS and chemotherapeutic agent 5-FU effectively induced apoptosis and genotoxicity and increased global DNA methylation in HT-29 cells. n contrast, Caco-2 cells showed no significant changes in apoptosis, cell migration, or global methylation following the same combined treatment. In addition, DNMT1 and VEGFA expressions remained unaltered in both Caco-2 and HT-29 cells. These findings suggest that the Caco-2 cell line exhibits greater resistance to these treatments, potentially due to its inherently high expression of efflux glycoproteins in the cell membrane. We provide evidence that DADS combined with 5-FU is potentially useful in the therapy of CRC. Overall, our findings highlight the importance of considering the distinct molecular and phenotypic characteristics of each tumor cell line in the investigation and development of new therapeutic strategies.

## Data Availability

The raw data supporting the conclusions of this article will be made available by the authors on request.

## Abbreviations

The following abbreviations are used in this manuscript:

CRC	Colorectal cancer
DADS	Diallyl disulfide
DNMT1	DNA Methyltransferase 1
HUVEC	Human umbilical vein endothelial cell
MMS	Methyl methanesulfonate
PBS	Phosphate-buffered saline
RS	Reactive species
VEGFA	Vascular endothelial growth factor A
5-FU	5-Fluorouracil

## References

[1] Li Q., Xia C., Li H., Yan X., Yang F., Cao M., Zhang S., Teng Y., He S., Cao M. (2024). Disparities in 36 Cancers across 185 Countries: Secondary Analysis of Global Cancer Statistics. Front. Med..

[2] Dekker E., Tanis P.J., Vleugels J.L.A., Kasi P.M., Wallace M.B. (2019). Colorectal Cancer. Lancet.

[3] McQuade R.M., Stojanovska V., Bornstein J.C., Nurgali K. (2017). Colorectal Cancer Chemotherapy: The Evolution of Treatment and New Approaches. Curr. Med. Chem..

[4] Chhikara B.S., Parang K. (2023). Global Cancer Statistics 2022: The Trends Projection Analysis. Chem. Biol. Lett..

[5] Thanikachalam K., Khan G. (2019). Colorectal Cancer and Nutrition. Nutrients.

[6] Rawla P., Sunkara T., Barsouk A. (2019). Epidemiology of Colorectal Cancer: Incidence, Mortality, Survival, and Risk Factors. Prz. Gastroenterol..

[7] Xu L., Sun J., Guo J., Guo S., Li J., Tang Y., Liu X. (2024). Transcriptional Factor KLF9 Overcomes 5-Fluorouracil Resistance in Breast Cancer via PTEN-Dependent Regulation of Aerobic Glycolysis. J. Chemother..

[8] Pachauri A., Chitme H., Visht S., Chidrawar V., Mohammed N., Abdel-Wahab B.A., Khateeb M.M., Habeeb M.S., Orabi M.A.A., Bakir M.B. (2023). Permeability-Enhanced Liposomal Emulgel Formulation of 5-Fluorouracil for the Treatment of Skin Cancer. Gels.

[9] Ramaswamy A., Bhargava P., Dubashi B., Gupta A., Kapoor A., Srinivas S., Shetty O., Jadhav P., Desai V., Noronha V. (2024). Docetaxel-Oxaliplatin-Capecitabine/5-Fluorouracil (DOX/F) Followed by Docetaxel versus Oxaliplatin-Capecitabine/5-Fluorouracil (CAPOX/FOLFOX) in HER2-Negative Advanced Gastric Cancers. JNCI Cancer Spectr..

[10] Burtness B., Harrington K.J., Greil R., Soulières D., Tahara M., De Castro G., Psyrri A., Basté N., Neupane P., Bratland Å. (2019). Pembrolizumab Alone or with Chemotherapy versus Cetuximab with Chemotherapy for Recurrent or Metastatic Squamous Cell Carcinoma of the Head and Neck (KEYNOTE-048): A Randomised, Open-Label, Phase 3 Study. Lancet.

[11] Vodenkova S., Buchler T., Cervena K., Veskrnova V., Vodicka P., Vymetalkova V. (2020). 5-Fluorouracil and Other Fluoropyrimidines in Colorectal Cancer: Past, Present and Future. Pharmacol. Ther..

[12] Wu H., Du J., Li C., Li H., Guo H., Li Z. (2022). Kaempferol Can Reverse the 5-Fu Resistance of Colorectal Cancer Cells by Inhibiting PKM2-Mediated Glycolysis. Int. J. Mol. Sci..

[13] Sethy C., Kundu C.N. (2021). 5-Fluorouracil (5-FU) Resistance and the New Strategy to Enhance the Sensitivity against Cancer: Implication of DNA Repair Inhibition. Biomed. Pharmacother..

[14] Chopra A.S., Lordan R., Horbańczuk O.K., Atanasov A.G., Chopra I., Horbańczuk J.O., Jóźwik A., Huang L., Pirgozliev V., Banach M. (2022). The Current Use and Evolving Landscape of Nutraceuticals. Pharmacol. Res..

[15] Ozalp Unal D., Sel T. (2024). Investigation of Antiproliferative Effects of Combinations of White and Black Garlic Extracts with 5-Fluorouracil (5-FU) on Caco-2 Colorectal Adenocarcinoma Cells. Mol. Nutr. Food Res..

[16] Mondal A., Banerjee S., Bose S., Mazumder S., Haber R.A., Farzaei M.H., Bishayee A. (2022). Garlic Constituents for Cancer Prevention and Therapy: From Phytochemistry to Novel Formulations. Pharmacol. Res..

[17] Machado A.R.T., Tuttis K., Santos P.W.d.S., Aissa A.F., Antunes L.M.G. (2022). Diallyl Disulfide Induces Chemosensitization to Sorafenib, Autophagy, and Cell Cycle Arrest and Inhibits Invasion in Hepatocellular Carcinoma. Pharmaceutics.

[18] Bal-Price A., Coecke S., Aschner M., Suñol C., Bal-Price A. (2011). Guidance on Good Cell Culture Practice (GCCP). Cell Culture Techniques.

[19] Page B., Page M., Noel C. (1993). A New Fluorometric Assay for Cytotoxicity Measurements In-Vitro. Int. J. Oncol..

[20] Bourgine J., Billaut-Laden I., Happillon M., Lo-Guidice J.-M., Maunoury V., Imbenotte M., Broly F. (2012). Gene Expression Profiling of Systems Involved in the Metabolism and the Disposition of Xenobiotics: Comparison between Human Intestinal Biopsy Samples and Colon Cell Lines. Drug Metab. Dispos..

[21] Mouradov D., Sloggett C., Jorissen R.N., Love C.G., Li S., Burgess A.W., Arango D., Strausberg R.L., Buchanan D., Wormald S. (2014). Colorectal Cancer Cell Lines Are Representative Models of the Main Molecular Subtypes of Primary Cancer. Cancer Res..

[22] Berg K.C.G., Eide P.W., Eilertsen I.A., Johannessen B., Bruun J., Danielsen S.A., Bjørnslett M., Meza-Zepeda L.A., Eknæs M., Lind G.E. (2017). Multi-Omics of 34 Colorectal Cancer Cell Lines—A Resource for Biomedical Studies. Mol. Cancer.

[23] Altonsy M.O., Madkour H., Yousef R., Andrews S. (2013). Diallyl Disulfide, from Garlic Oil, Synthesizes Human Colonic Adenocarcinoma Cell Line (Caco-2) to TNF-Alpha- Mediated Apoptosis through up-Regulation of Membrane FAS Levels. Am. J. Sci..

[24] Marni R., Kundrapu D.B., Chakraborti A., Malla R. (2022). Insight into Drug Sensitizing Effect of Diallyl Disulfide and Diallyl Trisulfide from *Allium sativum* L. on Paclitaxel-Resistant Triple-Negative Breast Cancer Cells. J. Ethnopharmacol..

[25] Mitra S., Das R., Emran T.B., Labib R.K., Noor-E-Tabassum, Islam F., Sharma R., Ahmad I., Nainu F., Chidambaram K. (2022). Diallyl Disulfide: A Bioactive Garlic Compound with Anticancer Potential. Front. Pharmacol..

[26] Germain E., Auger J., Ginies C., Siess M.-H., Teyssier C. (2002). In Vivo Metabolism of Diallyl Disulphide in the Rat: Identification of Two New Metabolites. Xenobiotica.

[27] Altonsy M.O., Andrews S.C. (2011). Diallyl Disulphide, a Beneficial Component of Garlic Oil, Causes a Redistribution of Cell-Cycle Growth Phases, Induces Apoptosis, and Enhances Butyrate-Induced Apoptosis in Colorectal Adenocarcinoma Cells (HT-29). Nutr. Cancer.

[28] Huang Y.-S., Xie N., Su Q., Su J., Huang C., Liao Q.-J. (2011). Diallyl Disulfide Inhibits the Proliferation of HT-29 Human Colon Cancer Cells by Inducing Differentially Expressed Genes. Mol. Med. Rep..

[29] Onar O., Telkoparan-Akillilar P., Yildirim O. (2023). Clitocybe Nebularis Extract and 5-fluorouracil Synergistically Inhibit the Growth of HT-29 Colorectal Cancer Cells by Inducing the S Phase Arrest. 3 Biotech..

[30] Yıldız F., Eciroğlu H., Suat Övey İ., Avnioğlu S. (2023). Effect of Combination Treatment of Protocatechuic Acid with 5-Fluorouracil and Oxaliplatin on Colon Cancer Caco-2 Cell Line. IJEB.

[31] Thejass P., Kuttan G. (2007). Inhibition of Angiogenic Differentiation of Human Umbilical Vein Endothelial Cells by Diallyl Disulfide (DADS). Life Sci..

[32] Rashidi M., Mohammadzadeh G., Sanaei A. (2021). Quercetin Synergistically Potentiates the Anti-Angiogenic Effect of 5- Fluorouracil on HUVEC Cell Line. Preprints.

[33] Wu X.-J., Kassie F., Mersch-Sundermann V. (2005). The Role of Reactive Oxygen Species (ROS) Production on Diallyl Disulfide (DADS) Induced Apoptosis and Cell Cycle Arrest in Human A549 Lung Carcinoma Cells. Mutat. Res./Fundam. Mol. Mech. Mutagen..

[34] Song J.-D., Lee S.K., Kim K.M., Park S.E., Park S.-J., Kim K.H., Ahn S.C., Park Y.C. (2009). Molecular Mechanism of Diallyl Disulfide in Cell Cycle Arrest and Apoptosis in HCT-116 Colon Cancer Cells. J. Biochem. Mol. Toxicol..

[35] Ozturk R.Y., Cakir R. (2024). In Vitro Anticancer Efficacy of Calendula Officinalis Extract-Loaded Chitosan Nanoparticles against Gastric and Colon Cancer Cells. Drug Dev. Ind. Pharm..

[36] Matuo R., Sousa F.G., Escargueil A.E., Grivicich I., Garcia-Santos D., Chies J.A.B., Saffi J., Larsen A.K., Henriques J.A.P. (2009). 5-Fluorouracil and Its Active Metabolite FdUMP Cause DNA Damage in Human SW620 Colon Adenocarcinoma Cell Line. J. Appl. Toxicol..

[37] Morana O., Wood W., Gregory C.D. (2022). The Apoptosis Paradox in Cancer. Int. J. Mol. Sci..

[38] Jakubíková J., Sedlák J. (2006). Garlic-Derived Organosulfides Induce Cytotoxicity, Apoptosis, Cell Cycle Arrest and Oxidative Stress in Human Colon Carcinoma Cell Lines. Neoplasma.

[39] Fouad M.A., Salem S.E., Hussein M.M., Zekri A.R.N., Hafez H.F., El Desouky E.D., Shouman S.A. (2018). Impact of Global DNA Methylation in Treatment Outcome of Colorectal Cancer Patients. Front. Pharmacol..

[40] Akone S.H., Ntie-Kang F., Stuhldreier F., Ewonkem M.B., Noah A.M., Mouelle S.E.M., Müller R. (2020). Natural Products Impacting DNA Methyltransferases and Histone Deacetylases. Front. Pharmacol..

[41] Druesne N., Pagniez A., Mayeur C., Thomas M., Cherbuy C., Duée P.-H., Martel P., Chaumontet C. (2004). Diallyl Disulfide (DADS) Increases Histone Acetylation and P21 Waf1/Cip1 Expression in Human Colon Tumor Cell Lines. Carcinogenesis.

[42] Sarabi M.M., Naghibalhossaini F. (2015). Association of DNA Methyltransferases Expression with Global and Gene-Specific DNA Methylation in Colorectal Cancer Cells. Cell Biochem. Funct..

[43] Scott A., Song J., Ewing R., Wang Z. (2014). Regulation of Protein Stability of DNA Methyltransferase 1 by Post-Translational Modifications. ABBS.

[44] Zhao S., Cui H., Fang X., Xia W., Tao C., Li J. (2024). Increased DNMT1 Acetylation Leads to Global DNA Methylation Suppression in Follicular Granulosa Cells during Reproductive Aging in Mammals. BMC Genom..

[45] Lee G.E., Kim J.H., Taylor M., Muller M.T. (2010). DNA Methyltransferase 1-Associated Protein (DMAP1) Is a Co-Repressor That Stimulates DNA Methylation Globally and Locally at Sites of Double Strand Break Repair. J. Biol. Chem..

[46] Elliott E.N., Sheaffer K.L., Kaestner K.H. (2016). The ‘de Novo’ DNA Methyltransferase Dnmt3b Compensates the Dnmt1-Deficient Intestinal Epithelium. eLife.

[47] Van Rijnsoever M., Elsaleh H., Joseph D., McCaul K., Iacopetta B. (2003). CpG Island Methylator Phenotype Is an Independent Predictor of Survival Benefit from 5-Fluorouracil in Stage III Colorectal Cancer. Clin. Cancer Res..

[48] Geißler A.-L., Geißler M., Kottmann D., Lutz L., Fichter C.D., Fritsch R., Weddeling B., Makowiec F., Werner M., Lassmann S. (2017). ATM Mutations and E-Cadherin Expression Define Sensitivity to EGFR-Targeted Therapy in Colorectal Cancer. Oncotarget.

[49] Gallois C., Laurent-Puig P., Taieb J. (2016). Methylator Phenotype in Colorectal Cancer: A Prognostic Factor or Not?. Crit. Rev. Oncol./Hematol..

